# CUL4-DDB1-CDT2 E3 Ligase Regulates the Molecular Clock Activity by Promoting Ubiquitination-Dependent Degradation of the Mammalian CRY1

**DOI:** 10.1371/journal.pone.0139725

**Published:** 2015-10-02

**Authors:** Xin Tong, Deqiang Zhang, Anirvan Guha, Blake Arthurs, Victor Cazares, Neil Gupta, Lei Yin

**Affiliations:** Department of Molecular & Integrative Physiology, University of Michigan Medical School, Ann Arbor, Michigan, United States of America; University of Pittsburgh, UNITED STATES

## Abstract

The CUL4-DDB1 E3 ligase complex serves as a critical regulator in various cellular processes, including cell proliferation, DNA damage repair, and cell cycle progression. However, whether this E3 ligase complex regulates clock protein turnover and the molecular clock activity in mammalian cells is unknown. Here we show that CUL4-DDB1-CDT2 E3 ligase ubiquitinates CRY1 and promotes its degradation both *in vitro* and *in vivo*. Depletion of the major components of this E3 ligase complex, including *Ddb1*, *Cdt2*, and Cdt2-cofactor *Pcna*, leads to CRY1 stabilization in cultured cells or in the mouse liver. CUL4A-DDB1-CDT2 E3 ligase targets lysine 585 within the C-terminal region of CRY1 protein, shown by the CRY1 585KA mutant’s resistance to ubiquitination and degradation mediated by the CUL4A-DDB1 complex. Surprisingly, both depletion of *Ddb1* and over-expression of Cry1-585KA mutant enhance the oscillatory amplitude of the *Bmal1* promoter activity without altering its period length, suggesting that CUL4A-DDB1-CDT2 E3 targets CRY1 for degradation and reduces the circadian amplitude. All together, we uncovered a novel biological role for CUL4A-DDB1-CDT2 E3 ligase that regulates molecular circadian behaviors via promoting ubiquitination-dependent degradation of CRY1.

## Introduction

The mammalian circadian clock functions to coordinate metabolic processes with light and food availability [1–3]. The current circadian model suggests that the circadian clock is mainly driven by an interlocked transcription-translation feedback loop [4, 5]. Within this model, the two positive regulators, BMAL1 and CLOCK, activate the expression of the negative regulators including *Cryptochrome* (*Cry1*, *Cry2*), *Period* (*Per1*, *Per2*, and *Per3*), and *Rev-erbα* [6, 7]. In turn, CRYs then hetero-dimerize with PERs and translocate back into the nucleus where they directly bind to BMAL1/CLOCK to repress their own transcription [8, 9].

Ubiquitination and proteasome-dependent degradation has been demonstrated to control the protein abundance of key clock proteins such as Rev-erbα, PER2, CRY1 and CRY2 [10]. In the case of CRY1 protein degradation, F-box leucine-rich repeat protein 3 (FBXL3) decreases the stability of CRY proteins [11]. Two FBXL3 mutant mice (C358S or I364T) showed defects in degrading CRY1 protein along with lengthened circadian period [11–13]. Another study revealed that AMP-activated kinase (AMPK) modulates the peripheral clock activity by triggering phosphorylation-dependent CRY1 degradation in response to nutrient signals [14]. Most recent reports show that another F-box E3 ligase, FBXL21, competes with FBXL3 to protect CRY1 from the ubiquitin-dependent degradation by proteasomes [15, 16]. We previously identified USP2a as a CRY1-specfic deubiquitinating enzyme to stabilize CRY1 protein and enhance its repression of circadian target genes in response to serum shock or TNF-α stimulation in hepatocytes [17]. Taken together, these studies suggest that CRY1 protein is subjected to ubiquitination catalyzed by multiple ubiquitination regulators in response to certain circadian cues.

In *Arabidopsis*, the damaged DNA-binding protein 1-Cullin 4A (DDB1-CUL4A) E3 ligase complex has been shown to mediate light-induced degradation of CRY via the substrate receptor COP1[18]. This regulation is also conserved in *Drosophila*, in which DDB1-CUL4A/Ramshackle (Brwd3) E3 ligase is involved in light-induced ubiquitination and degradation of *Drosophila* CRY [19]. DDB1 was initially identified as a critical component of the damaged DNA protein complex in the response to UV exposure. Later, it has been shown to function as a linker protein in the DDB1-CUL4A E3 ligase complex by interacting with CUL4A through its seven bladed β-propeller-B (BP-B) domain, and with CUL4A-associated proteins (DCAFs) through its BP-A and BP-C domains [20]. DDB1-CUL4A E3 complex has been shown to ubiquitinate a wide range of protein substrates such as histone H2A, CDT1, Myc, p27, and p21 for degradation [21–27]. *In vivo*, *Ddb1* global and brain-specific knockout mice are embryonic lethal, possibly due to cell cycle dysregulation [28]. Loss of *Ddb1* in the mouse liver causes liver cancer at the age of 17 months [29]. So far, whether and how DDB1-CUL4A E3 ligase regulates the mammalian CRY1 protein stability and CRY1-mediated circadian activity remains unclear.

Here we report that DDB1-CUL4A E3 ligase interacts with the mammalian CRY1 and promotes its degradation via ubiquitination in both hepatocytes and the liver. Hepatic *Ddb1* deficiency or depletion greatly reduces CRY1 ubiquitination and promotes its protein stabilization throughout the circadian cycle. Furthermore, we mapped lysine 585 of CRY1 as the ubiquitination site targeted by DDB1-CUL4A E3 ligase and revealed that the CRY1 K585A mutant is resistant to ubiquitination and degradation. We identified CDT2 (Cdt10-dependent transcript 2, also named DCAF2) as the CRY1-binding DCAF protein and showed that the CDT2-cofactor PCNA (proliferating cell nuclear antigen) is required for CRY1 degradation. In conclusion, our study revealed that the CUL4A-DDB1-CDT2 E3 ligase regulates circadian clock oscillations by degrading the mammalian CRY1 protein via ubiquitination.

## Materials and Methods

### Reagents and plasmids

The full-length Ddb1 and Cul4A expression vectors were purchased from the Open Biosystems (Thermo Scientific) and then subcloned into the pQCXIP vector (Clontech). *Cry1* mutants were generated using the QuikChange site-direct mutagenesis kit (Agilent). *Ddb1* and *Cul4a* shRNA knockdown constructs were made by ligating the targeting oligo sequences into the RNAi-Ready pSIREN-Retro-Q vector (Clontech). The *mPer2* promoter-driven luciferase reporter construct is a generous gift from Dr. Hogenesch at University of Pennsylvania. The Cdt2 expression construct and specific antibody were kindly provided by Dr. Anindya Dutta at University of Virginia. All plasmids were confirmed by automated sequencing analysis. All the commercial antibodies used in this work were: anti-CRY1(Santa Cruz Biotechnology SC-101006), anti-FLAG (Sigma F1804), anti-ubiquitin (Sigma U5319), and anti-MYC (Bethyl A190-105A). Anti-M2 agarose beads (A2220) were also purchased from Sigma. MG132 was purchased from Biomol (Plymouth, PA).

### Animal care and adenoviral tail vein injection in mice

All animal care and use procedures described in this study were approved by the University of Michigan Institutional Animal Care and Use Committee (IACUC). All the procedures were performed in accordance with guidelines of IACUC. WT C57BL/6J male mice (8 to 10 wks) were maintained on a 12:12 LD cycle with free access to standard diet and water. *Ddb1* liver-specific knockout (*Ddb1-LKO*) mice were generated by crossing *Ddb1* ^*flox/flox*^ mice (provided by Dr. Yong Cang at the Sanford-Burnham Medical Research Institute) with Alb-Cre transgenic mice. Genotypes were confirmed by PCR reactions. In all subsequent experiments, *Ddb1* ^*flox/flox*^ littermates without the *Cre* transgene were used as controls. For adenoviral injections, 1x10^12^ plaque-forming units (*pfu*) per recombinant adenovirus were administrated via tail-vein injection. For each virus, a group of 4 to 5 mice were injected with the same dose treatment. 10 days after injection, mice were sacrificed at ZT8 after overnight fasting and liver tissues were harvested for protein analysis.

### *In vitro* ubiquitination assay

The GST-CRY1-WT and GST-CDT1 substrates were captured and eluted off of GST-agarose beads (Sigma) after IPTG induction in transformed BL21 cells for 3 hr at 37°C. The FLAG-tagged DDB1 and CUL4A E3 complexes were immunoprecipitated from U2OS cells by anti-FLAG M2 antibody (Sigma) after Ad-Ddb1/Cul4A transduction. Ad-GFP transduced cells were used as IP control. Protein-A sepharose beads were equilibrated in ligase assay buffer (25 mM Tris-HCl pH = 7.5, 50 mM NaCl, 1 mM EDTA, 0.01% NP-40, 10% glycerol) twice and mixed with the GST substrate in 30 μL of the ubiquitin mix containing: 25 mM Tris-HCl, pH = 7.4, 5 mM MgCl_2_, 2 mM ATP, 2 mM sodium fluoride, 1mM DTT, 10 nM okadaic acid, 250 μM ubiquitin aldehyde (Boston Biochem), 120 ng E1 (His_6_-UBE1, Boston Biochem), 300 ng E2 (UbcH5a, Boston Biochem), and 10 μg ubiquitin (Boston Biochem) [22]. The reactions were kept in a 37°C shaker for 2 hr and then denatured by adding 5X SDS loading buffer and boiling at 95°C for 5 mins. The final reactions were run in an 8% SDS-PAGE gel and subjected to immunoblotting with anti-ubiquitin antibody.

### Serum shock and synchronization study

Hepa1c1c-7 cells were used in the synchronization study. The confluent cells were transduced with Ad-shLacZ *vs*. Ad-shDdb1 or transfected with Cry1-WT *vs*. Cry1-585KA. 24 hr later, 50% horse serum was added as described previously [30]. Cells then were collected for protein analysis at 4-hr intervals between 16 hr and 60 hr time points.

### Real-time bioluminescence assay

U2OS-*Bmal1*-*luc* cells [31] were kindly provided by Dr. John Hogenesch from University of Pennsylvania and maintained in DMEM with 5% FBS and 1 μg/mL puromycin. Manipulation of Cry1 expression was achieved by transient transfection with the expression vector for *Cry1* over-expression or *shRNA* knockdown. Two days post-transfection, the medium was changed to phenol red-free DMEM containing 5% FBS, NEAA, 1X penicillin/streptomycin/glutamine, 20 mM HEPES, 0.1 mM Luciferin (Promega), 100 nM dexamethasome, and 10 μM forskolin for synchronization. The dishes were covered with sterile glass coverslips, sealed with sterile vacuum grease, and placed into the LumiCycle (Actimetrics). Bioluminescence levels were measured every 10 min for 5 days or more. The data analysis was performed using the Lumicycle Analysis program (Periodogram graph, poly order 5) and means of both amplitude and period were extracted and exported for statistical analysis

### Confocal Imaging

U2OS cells were plated onto glass-bottom micro-well dishes (MatTek Corp. P35G-1.5-14-C) and transfected with mCherry-Cry1 and GFP-Cdt2 expression constructs. Cells were fixed in ice-cold absolute methanol for 10 minutes and then permeabilized by adding 0.25% Triton X-100 on ice for 6 min. Nuclei were stained by 20 mg/L DAPI solution at room temperature for 6 min. Cells were mounted on an Olympus IX81 microscope with a Plan-Apochromat 200x 1.4 NA oil immersion objective. Images were acquired by an EM-CCD Hamamatsu camera (ImageEM) with the following filter sets: Ex 472/30; 562/40 and EM 520/35; 641/75 for GFP and RFP. Fluorescent images were adjusted for brightness and contrast, pseudo-colored, and merged using Image J software (http://imagej.nih.gov/ij/).

## Results

### CUL4A-DDB1 E3 ligase promotes degradation of the mammalian CRY1 protein

**Fig 1:**
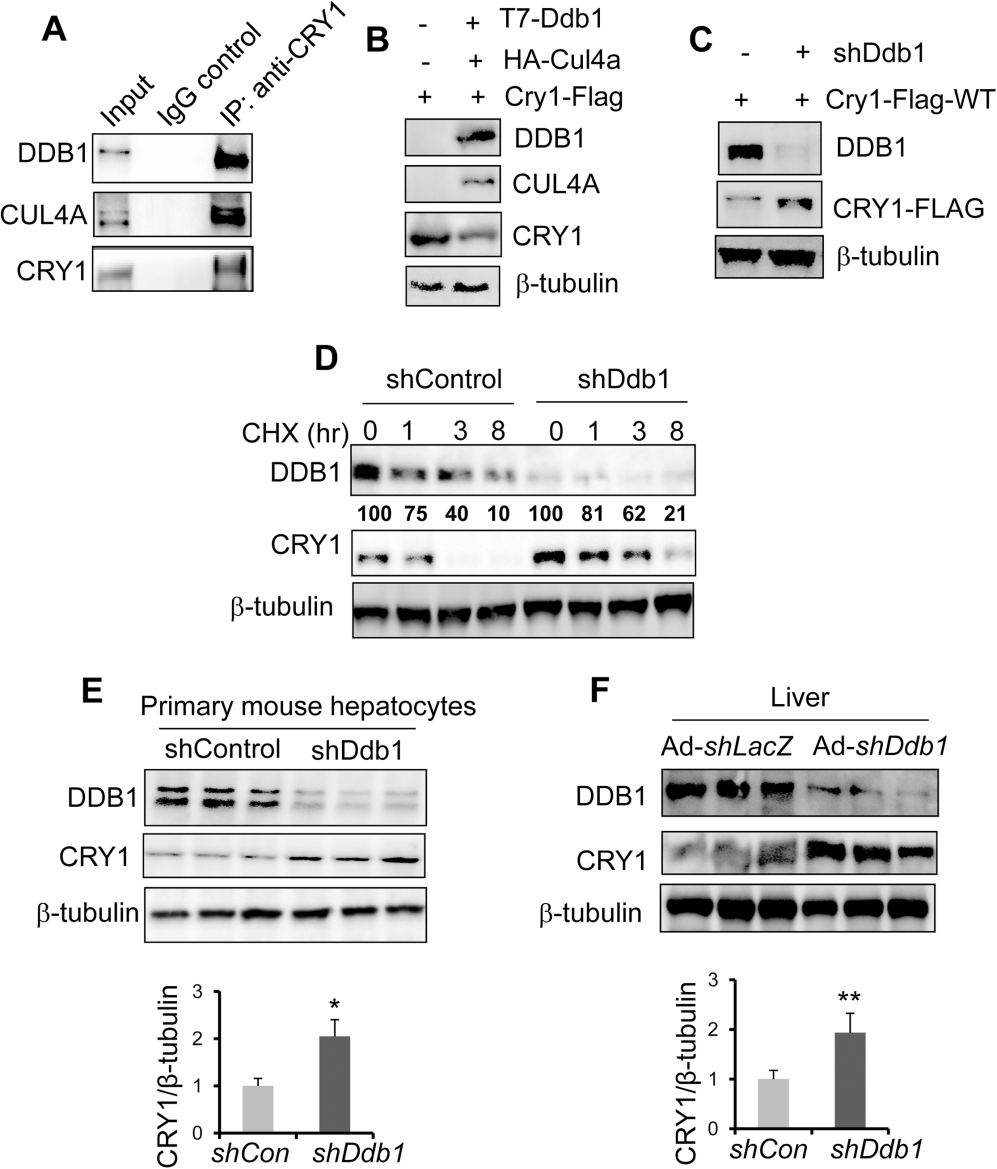
CUL4A-DDB1 E3 ligase promotes CRY1 protein degradation in cells and the mouse liver. **(A)** Endogenous CRY1 interacts with DDB1-CUL4A E3 ligase complex. U2OS cells were first synchronized by serum shock to elevate the endogenous CRY1 protein expression. Cells were then treated with MG132 for 8 hr and harvested to detect CRY1 interaction with DDB1-CUL4A E3 ligase. About 5 mg of total nuclear protein were incubated with anti-CRY1 (1:100) for 16 hr at 4C. The presence of CRY1 and DDB1 complex were detected by using antibodies against CUL4A, DDB1, and CRY1. The representative one of three individual IP experiments is shown here. (**B**) Overexpression of DDB1 and CUL4A reduces the CRY1 abundance in 293T cells. 293T cells were co-transfected with Cry1-Flag along with either GFP control or a mixture of Ddb1 and Cul4A. Cells were collected 48 hr later to examine the expression of CRY1-FLAG protein by immunoblotting. (**C**) Depletion of *Ddb1* elevates the expression of CRY1 in 293T cells. 293T cells were co-transfected with Cry1-Flag along with either control shRNA or *Ddb1* shRNA. Cells were collected 48 hr later for examining CRY1-FLAG protein by immunoblotting. (**D**) *Ddb1* knockdown increases CRY1 protein stability in 293T cells. 293T cells were co-transfected with Cry1-Flag along with either control shRNA or shDdb1 for 48 hr. Cells were then treated with cycloheximide (CHX, 100 μg/mL) for 0, 1, 3, and 8 hr before harvest. The expression levels of CRY1-FLAG were determined by immunoblotting. A representative of three individual experiments was shown here. The expression of CRY1-FLAG was quantified and normalized to loading control ß-tubulin. **(E)** Acute *Ddb1* knockdown increases CRY1 protein in primary mouse hepatocytes. Primary mouse hepatocytes were transduced with either Ad-shLacZ or Ad-shDdb1 and then isolated after 48 h for CRY1 and DDB1 protein expression by immunoblotting. The expression of CRY1-FLAG was quantified, normalized to loading control ß-tubulin, and plotted as mean ± S.D. (n = 3). *p-value < 0.05 by the Student’s *t-*test. (**F**) Liver-specific acute knockdown of *Ddb1* augments the CRY1 protein expression in the liver. Mice of 8–10 weeks were injected with either Ad-shLacZ or Ad-shDdb1 via the tail vein. Three weeks later, liver CRY1 protein levels were detected by immunoblotting with anti CRY1 (n = 3). The knockdown effect of Ad-shDdb1 was verified by the levels of DDB1 in the same samples. The expression of CRY1-FLAG was quantified, normalized to loading control ß-tubulin, and plotted as mean ± S.D. (n = 3). *p-value < 0.05 by the Student’s *t-*test.

*Drosophila* CUL4A-DDB1-BRWD3 E3 ligase has been shown to be involved in light-induced ubiquitination and degradation of the fly CRY protein [19]. The mammalian CRY1 protein shares 48% sequence identity with its *Drosophila* homologue [32], raising the possibility of a conserved regulatory mechanism through evolution. To determine whether DDB1-CUL4A E3 ligase could target the mammalian CRY1 for degradation, we first looked into a possible physical interaction between CRY1 and DDB1-CUL4A E3 ligase complex after proteasome inhibition. We chose U2OS cells for this investigation due to the high basal expression of both DDB1 and CUL4A proteins. Indeed, in U2OS cells treated with proteasome inhibitor MG132, we detected a protein complex containing the endogenous CRY1, DDB1, and CUL4A by a co-immunoprecipitation (co-IP) assay with anti-CRY1 and immunoblotting with respective antibodies (**Fig 1A**). Next, we examined how manipulation of CUL4A-DDB1 E3 ligase affects the protein abundance of CRY1. Indeed, we found that over-expression of CUL4A and DDB1 markedly reduces the CRY1 protein expression (**Fig 1B**). In contrast, disruption of this E3 ligase via knockdown of *Ddb1* by shRNA greatly increases CRY1 in 293T cells (**Fig 1C**). If the CUL4A-DDB1 activity does promote CRY1 protein degradation, we expect that the degradation rate of CRY1 would be reduced when this E3 ligase complex is inhibited. The protein half-life of CRY1 in the control group is less than 3 hr, whereas acute depletion of *Ddb1* by shRNA extends the CRY1 half-life beyond 8 hr after treatment of protein synthesis inhibitor cycloheximide (CHX) in 293T cells (**Fig 1D**).

To further address whether this regulation occurs in hepatocytes, we generated adenoviral shRNA against *Ddb1* (Ad-shDdb1) to knockdown *Ddb1* in the mouse primary hepatocyte and mouse liver. In *Ddb1*-depleted primary mouse hepatocytes, DDB1 protein is greatly reduced, whereas CRY1 protein shows a significant increase of about 2 fold **(Fig 1E).** In the mouse liver, injection of Ad-shDdb1 via tail vein leads to obliteration of DDB1 protein compared with Ad-shLacZ-injected control. In the same liver tissues, the CRY1 protein level was significantly elevated about 2 fold (**Fig 1F**). To summarize, our data clearly demonstrated that CUL4A-DDB1 E3 ligase promotes degradation of the CRY1 protein in hepatocytes and liver tissues.

### DDB1-CUL4A directly ubiquitinates CRY1 protein

**Fig 2:**
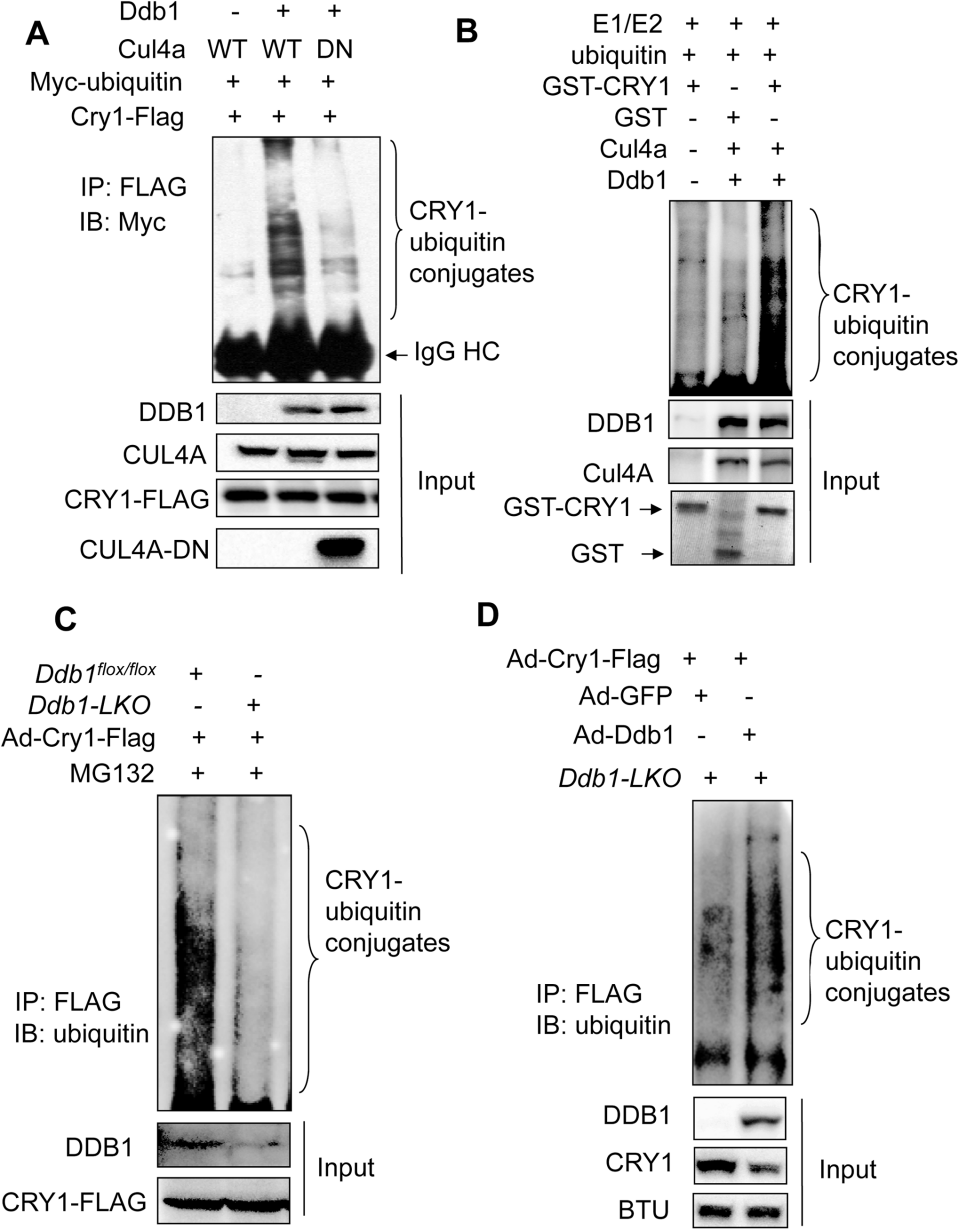
Cul4A-DDB1 E3 ligase promotes CRY1 ubiquitination both *in vivo* and *in vitro*. (**A**) Enhanced CRY1 ubiquitination in the presence of CUL4A-DDB1 E3 ligase. 293T cells were co-transfected with Cry1-Flag and Myc-ubiquitin in the presence of GFP, HA-Cul4A plus T7-Ddb1, or HA-Cul4A-DN plus T7-Ddb1. 24 hr after transfection, cells were treated with proteasome inhibitor MG132 (5 μM) for 16 hr and then harvested for detection of CRY1 ubiquitination. The protein levels of CRY1, CUL4A, DDB1 and DN-CUL4A in the whole cell lysates were determined by immunoblotting with specific antibodies. All the experiments were repeated at least three times and a representative result was shown here. (**B**) *In vitro* CRY1 ubiquitination by CUL4A-DDB1 E3 ligase. The purified GST-CRY1 fusion protein was mixed with the DDB1-CUL4A complex isolated from adenovirus-transduced 293T cells in the presence of E2, E3, and ubiquitin at 37°C for 2 hr before immunoblotting with anti-ubiquitin. GST-CRY1 and GST control were shown by Coomassie blue staining.(**C**) Reduced CRY1 ubiquitination in *Ddb1-LKO* primary mouse hepatocytes. After isolation from either 8-wk old *Ddb1* ^*flox/flox*^ or *Ddb1-LKO* mice, primary mouse hepatocytes were transduced with Ad-Cry1-Flag for 24 hr and treated with MG132 (5 μM) for 16 hr before harvest. The protein lysates were subjected to denaturing IP to detect CRY1 ubiquitination. The protein levels of CRY1 and DDB1 in the whole cell lysates were measured by immunoblotting with either anti-FLAG or anti-DDB1. All the experiments were repeated three times and the representative result was shown here. (**D**) Adenoviral overexpression of *Ddb1* rescues CRY1 ubiquitination in *Ddb1-LKO* primary mouse hepatocytes. Primary mouse hepatocytes were isolated from *Ddb1-LKO* mice and transduced by Ad-Cry1 along with either Ad-GFP or Ad-Ddb1. 24 hr post-transduction, cells were treated with MG132 for 8 hr and then harvested for detecting CRY1-ubiquitination.

To test whether DDB1-CUL4A promotes CRY1 protein degradation via direct ubiquitination, we performed a cell-based ubiquitination assay with over-expression of both DDB1 and CUL4A. The CRY1 poly-ubiquitin conjugates was detected in 293T cells over-expressing both wild-type (WT) CUL4A and the linker protein DDB1 but not in cells expressing a truncated (1-290aa) dominant negative (DN) form of CUL4A (**Fig 2A**), which fails to form a functional E3 ligase complex [33]. CRY1 ubiquitination by DDB1-CUL4A was also observed in mouse hepatoma Hepa1 cells and human osteosarcoma U2OS cells (**S1A and S1B Fig**), indicating that such regulation is conserved in multiple cell types. To determine whether the DDB1-CUL4A E3 ligase can directly ubiquitinate CRY1, we performed an *in vitro* ubiquitination assay using the CUL4A-DDB1 E3 ligase immunoprecipated from adenovirus-transduced 293T cells and GST-CRY1 fusion protein purified from BL21 competent cells along with E1, E2, and ubiquitin. As shown in **Fig 2B**, the CUL4A-DDB1 E3 ligase complex directly promotes ubiquitination of GST-CRY1.

FBXL3 has been identified as an E3 ligase of CRY1 to regulate circadian rhythms. To determine whether CRY1 ubiquitination by CUL4A-DDB1 E3 ligase is independent of FBXL3, we compared the ubiquintination response of both CRY1-WT and CRY1-S71A mutant, which was shown to be resistant to FBXL3-mediated degradation [14], in the presence of CUL4A-DDB1 E3 ligase. CUL4A-DDB1 E3 ligase ubiquitinates CRY1-WT and CRY1-S71A mutant equally in 293T cells **(S2A Fig)**. In addition, acute depletion of *Ddb1* also stabilizes the CRY1-S71A mutant in 293T cells **(S2B Fig)**, suggesting that CUL4A-DDB1 E3 ligase could be an independent E3 ligase complex targeting CRY1 for ubiquitination-dependent degradation.

To evaluate the relative contribution of DDB1-CUL4A E3 ligase in hepatic CRY1 ubiquitination, we transduced primary mouse hepatocytes from either control or hepatocyte-specific *Ddb1* knockout (*Ddb1-LKO*) mice with Ad-Cry1-Flag virus and compared the levels of CRY1-FLAG ubiquitin conjugates. As shown in **Fig 2C**, FLAG-tagged CRY1 ubiquitination is largely undetectable in PMHs isolated from *Ddb1-LKO* mice. However, restoring *Ddb1* by adenovirus enhanced the level of polyubiquitinated CRY1 conjugates in *Ddb1-LKO* PMHs **(Fig 2D).** These results indicate a critical role of DDB1-CUL4A E3 ligase in driving CRY1 ubiquitination and protein degradation in mouse hepatocytes.

### CUL4A-DDB1 E3 ligase targets C-terminal region of CRY1 protein

So far, we have shown that CUL4A-DDB1 ubiquitinates CRY1 and controls its stability and oscillations independently of FBXL3. The next important question was to identify the region within CRY1 protein directly targeted by CUL4A-DDB1. To this end, we generated a panel of C-terminal deletion mutants of CRY1 (**S3A Fig**) and compared their responses to over-expression of CUL4A and DDB1. Deletion of the last 100aa (CRY1-1-500aa) was sufficient to increase CRY1 protein stability (**S3B Fig**). Moreover, co-transfection of CUL4A-DN markedly stabilized CRY1-WT while showing a lesser effect on mutants (1-500aa and 1-300aa) (**S3C Fig**). Finally, the CRY1-1-500aa mutant was more stable than CRY1-WT at all time points in synchronized Hepa1 cells (**S3D Fig**). Thus, CUL4A-DDB1 is likely to target the last C-terminal region of CRY1 protein, while FBXL3 seems to target S71 at the N-terminal region of CRY1 [14].

### CUL4A-DDB1 targets Lysine 585 of CRY1 for ubiquitination

**Fig 3:**
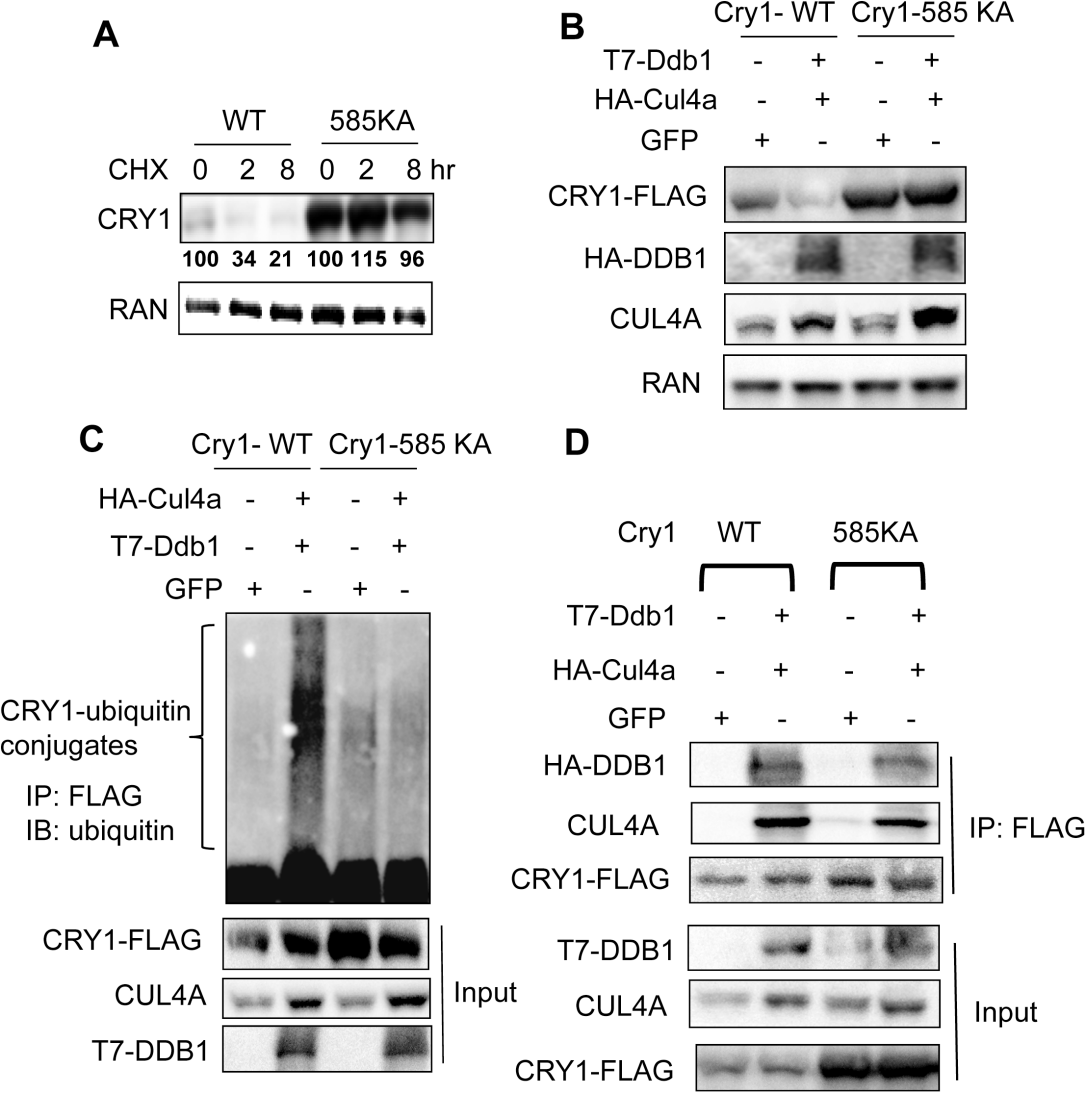
CUL4A-DDB1 E3 ligase targets the N-terminal lysine 585 of CRY1 for ubiquitination and degradation. **(A)** Comparison of the CRY1-585KA-Flag *vs*. CRY1-WT-Flag protein stability. 48 hr later after transfection with either Cry1-WT-Flag or Cry1-585KA-Flag, 293T cells were treated with CHX for 0, 2, and 8 hr before harvest. The expression levels of FLAG-tagged CRY1 were determined by immunoblotting by anti-FLAG, quantified and normalized by loading control control RAN. The percentage of the remaining CRY1 at each time point was calculated by comparing to the CRY1 abundance at time 0 hr. (**B**) CRY1-585KA-FLAG mutant is resistant to degradation triggered by DDB1-CUL4A E3 ligase. After transfection with Cry1-WT-Flag *vs*. Cry1-585KA-Flag along with GFP control or T7-Ddb1 plus HA-Cul4a, 293T cells were harvested 48 hr later for immunoblotting with anti-FLAG. (**C**) Cry1-585KA-FLAG mutant is resistant to ubiquitination mediated by DDB1-CUL4A E3 ligase. 293T cells were transfected with expression vectors encoding either Cry1-WT-Flag or Cry1-585KA-Flag in the presence of GFP control or T7-Ddb1 plus HA-Cul4a. Cells were then treated with MG132 for 16 hr and harvested for ubiquitination IP. The expression levels of CRY1-FLAG, DDB1 and CUL4A in the whole cell lysates were determined by immunoblotting. **(D)** CRY1-585KA mutant interacts with the CUL4A-DDB1 complex. 293T cells were co-transfected with Cry1-585KA-Flag *vs*. Cry1-WT-Flag in the presence or absence of HA-Cul4a plus T7-Ddb1. 24 hr post-transfection, cells were treated with MG132 for 16 hr and collected for co-IP with anti-FLAG. The presence of CRY1, CUL4A, and DDB1 in the immunocomplex was detected by antibodies against CUL4A, DDB1, and FLAG.

So far we have demonstrated that CUL4A-DDB1 is likely to ubiquitinate the C-terminal region of CRY1 protein. Several lysine residues are found within the last 100aa of the CRY1 N-terminus, including K571, K585, and K599. Among all the lysine to alanine CRY1 mutants, we found only CRY1 585KA to be stabilized with an extended half-life (more than 2 hr) in comparison with CRY1 WT in a CHX chase experiment in 293T cells (**Fig 3A**). More relevant to our study, CRY1 585KA is resistant to CUL4A-DDB1-mediated degradation and ubiquitination in 293T cells (**Fig 3B and 3C)**. To test whether stabilization of CRY1 585KA is due to its lost ability to interact with the CUL4A-DDB1 complex, we performed a co-IP assay after transient transfection with CRY1-WT or 585KA in the presence of Ddb1 and Cul4a overexpression. As shown in **Fig 3D**, CRY-585KA mutant retains as strong an interaction with DDB1 and CUL4A as CRY1-WT in 293T cells, suggesting that 585KA mutation blocks CRY1 ubiquitination without disrupting its interaction with DDB1-CUL4A. In summary, we identified a critical lysine residue within the C-terminal domain of CRY1 protein as the ubiquitination site targeted by DDB1-CUL4A E3 ligase.

### Identification of CDT2 as the CRY1-binding DCAF protein

CUL4A-DDB1 E3 ligase has been shown to interact with its targets through substrate-binding DCAF (DDB1-CUL4A associated factor) proteins, which share a conserved WD40 motif (**S4A Fig**) [33, 34]. A number of DCAFs have been identified via affinity purification and mass spectrometry analysis [33, 35]. To screen which known DCAF factor targets CRY1 protein degradation, we generated shRNA constructs targeting a panel of *Dcafs* (*Ddb2* and *DCAF1-17*) for their effects on the *Per2-luc* activity, which was shown to be repressed by either *Ddb1* knockdown or Cul4A-DN overexpression (**S4B and S4C Fig**). In a screen in 293T cells, we observed that only *Cdt2* (*Dcaf2*) and *Dcaf6* depletion reduces *Per2-luc* activity, whereas other DCAF shRNAs show either no effect or induction of *Per2-luc* (**S4D Fig**). We further demonstrated that only the shRNA targeting *Cdt2* but not *Dcaf6* significantly increases the CRY1 protein abundance in cells (**S4E Fig**).

**Fig 4:**
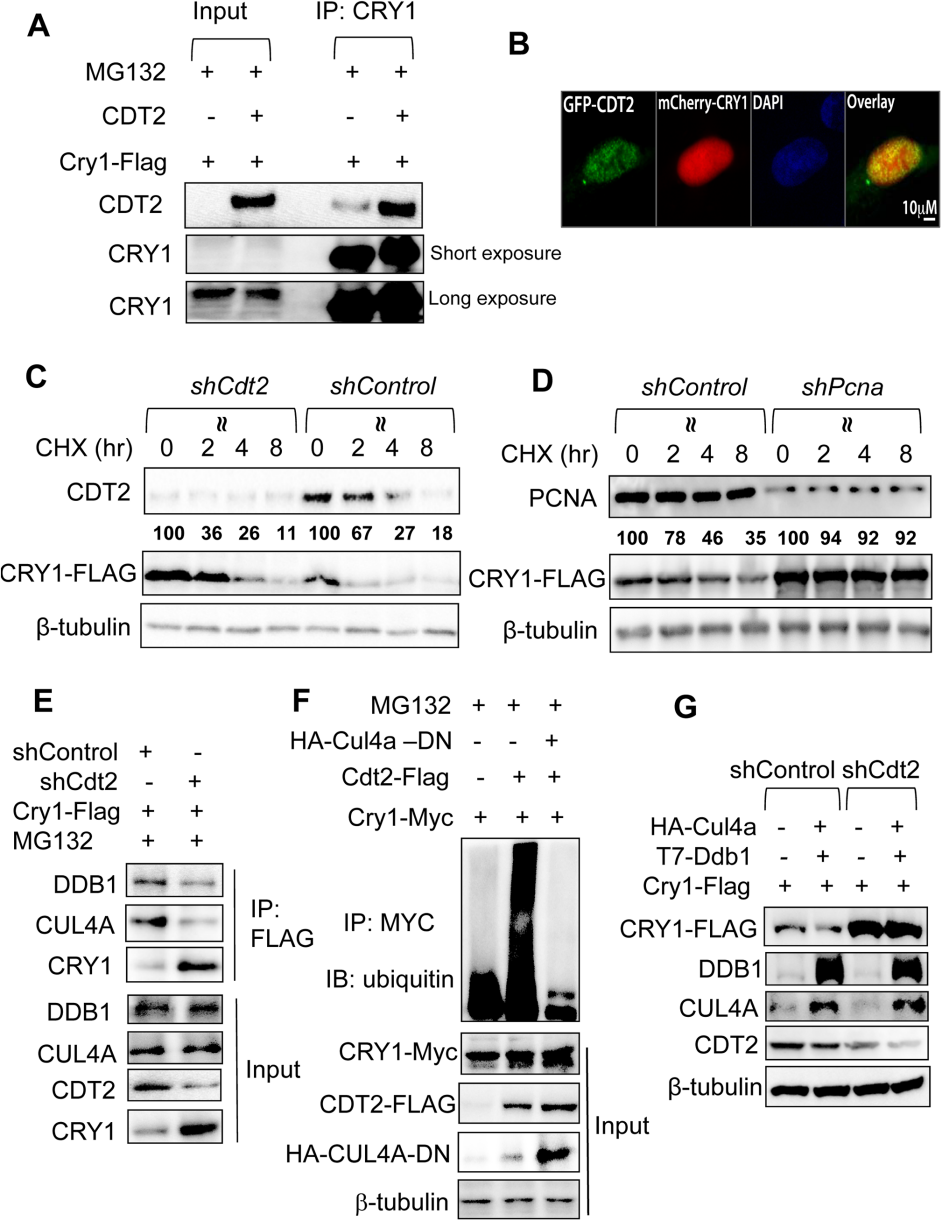
CDT2 functions as a substrate-binding protein for DDB1-CUL4A-mediated regulation of CRY1 protein. (**A**) CRY1 interacts with both the endogenous and overexpressed CDT2. U2OS cells were transfected with Cry1-Flag in the presence or absence of Cdt2 expression vector. Cells were treated with MG132 for 16 hr prior to IP with anti-CRY1. The presence of CDT2 was detected by anti-CDT2. (**B**) CRY1 and CDT2 co-localize in the nucleus. U2OS cells were transfected with both mCherry-Cry1 and GFP-Cdt2 and treated with MG132 at 10 μM for 3 hr prior to methanol fixing, Triton X-100 permeabilization, and DAPI counterstaining. mCherry-CRY1 and GFP-CDT2 fluorescent protein signals were captured by confocal imaging and overlaid by Image J software. (**C**) *Cdt2* knockdown increases CRY1 protein stability in 293T cells. 293T cells were co-transfected with Cry1-Flag along with either control shRNA or Cdt2 shRNA for 48 hr. Cells were then treated with cycloheximide (CHX) for 0, 2, 4, and 8 hr before harvest. The expression levels of CRY1-FLAG were determined by immunoblotting and quantified. The percentage of the remaining CRY1 at each time point was calculated by comparing to the CRY1 abundance at time 0 hr. The experiments were repeated three times and a representative one is shown here. **(D)** Effect of knockdown of CDT2 co-factor *Pcna* on the CRY1 protein degradation. 293T cells were co-transfected with CRY1-Flag along with either shControl or shPcna vector. 48 h later, Cells were treated with CHX at 10 μg/mL for the indicated times prior to immunoblotting**.** The percentage of the remaining CRY1 at each time point was calculated by comparing to the CRY1 abundance at time 0 hr. **(E)** Depletion of *Cdt2* reduces CRY1 interaction with DDB1-CUL4A complex. U2OS cells were transfected with CRY1-Flag together with either shControl or shCdt2 vectors. 48 hr later, cells were exposed to MG132 for 8 hr before harvest. The interaction between CRY1-FLAG and the endogenous DDB1-CUL4A complex was examined by immunoprecipitation with anti-FLAG**. (F)** CDT2 promotes CRY1 ubiquitination via a functional CUL4A-DDB1 complex. Myc-Cry1 was co-transfected with or without Cdt2 expression vector. To block the endogenous CUL4A-DDB1 complex, HA-Cul4a-DN was co-transfected along with Cry1 and Cdt2 vector. Cells were treated with MG132 for 16 hr before detection of CRY1 ubiquitination, as described above. **(G)** *Cdt2* depletion blocks on DDB1-CUL4A-mediated CRY1 degradation. U2OS cells were transfected with Cry1-Flag or Cry1-Flag/T7-Ddb1/HA-Cul4a in the presence of either shControl or shCdt2 vector. Cells were then harvested 48 hr later for examining levels of CRY1-FLAG, DDB1, CUL4A, CDT2, and β-tubulin.

Although well-known for its critical role in cell cycle regulation [36], DDB1-CUL4A-CDT2 E3 ligase has not been linked to the circadian clock. To firmly establish CDT2 as the CRY1-binding DCAF protein that links CRY1 and CUL4A-DDB1 E3 ligase, we first tested whether CRY1 interacts with CDT2 in U2OS cells transfected with Cdt2 and Cry1-Flag overexpression constructs after IP with anti-FLAG. As shown in **Fig 4A**, CRY1-FLAG indeed interacts with both the endogenous and overexpressed CDT2 in MG-132-treated U2OS cells. CDT2 is known for its nuclear localization [37], whereas CRY1 was reported to shuttle between the cytoplasm and the nucleus [9, 38]. To determine whether both CRY1 and CDT2 reside in the same subcellular compartment, we transfected U2OS cells with mCherry-Cry1 and GFP-Cdt2 expression constructs and found both fusion proteins to localize predominantly inside the nucleus after MG-132 treatment. Consistent with the co-IP results, overlay of confocal imaging revealed that mCherry-CRY1 and GFP-CDT2 co-localize in the nucleus (**Fig 4B**). Moreover, we observed a clear stabilization of CRY1-FLAG in a CHX chase experiment in *Cdt2*-depleted U2OS cells (**Fig 4C**). In the case of *Cdt2* depletion, the half-life of CRY1 protein is prolonged beyond 3 hr. Since CDT2 E3 ligase-mediated ubiquitination and degradation have been shown to require the binding of its substrates to PCNA [39, 40], we tested whether PCNA is also involved in CRY1 protein degradation. As shown in **Fig 4D**, *Pcna* depletion by shRNA almost completely blocks CRY1 protein degradation following CHX treatment in 293T cells. In summary, our results demonstrate that inhibition of CDT2 or its co-factor PCNA leads to stabilization of CRY1 protein.

To further test the role of CDT2 in DDB1-CUL4A E3-mediated CRY1 ubiquitination and degradation, we checked whether *Cdt2* knockdown impairs the complex formation between CRY1 and DDB1-CUL4A E3 ligase in U2OS cells. Indeed, acute depletion of *Cdt2* by shRNA greatly reduces the amount of DDB1 and CUL4A proteins interacting with CRY1 even though the overall CRY1 protein level is elevated upon *Cdt2* depletion **(Fig 4E).** Meanwhile, overexpression of *Cdt2* is sufficient to promote CRY1-Myc protein ubiquitination, whereas concomitant overexpression of HA-CUL4A-DN blocks its effect in U2OS cells (**Fig 4F).** Lastly, acute depletion of *Cdt2* by shRNA blocked CRY1 protein degradation promoted by DDB1-CUL4A over-expression in U2OS cells (**Fig 4G**). To summarize, we identified CDT2 as the major PCNA-dependent CRY1-binding DCAF for DDB1-CUL4A-mediated ubiquitination and degradation of CRY1.

### CUL4A-DDB1 E3 ligase regulates CRY1 protein oscillations and the circadian clock

**Fig 5:**
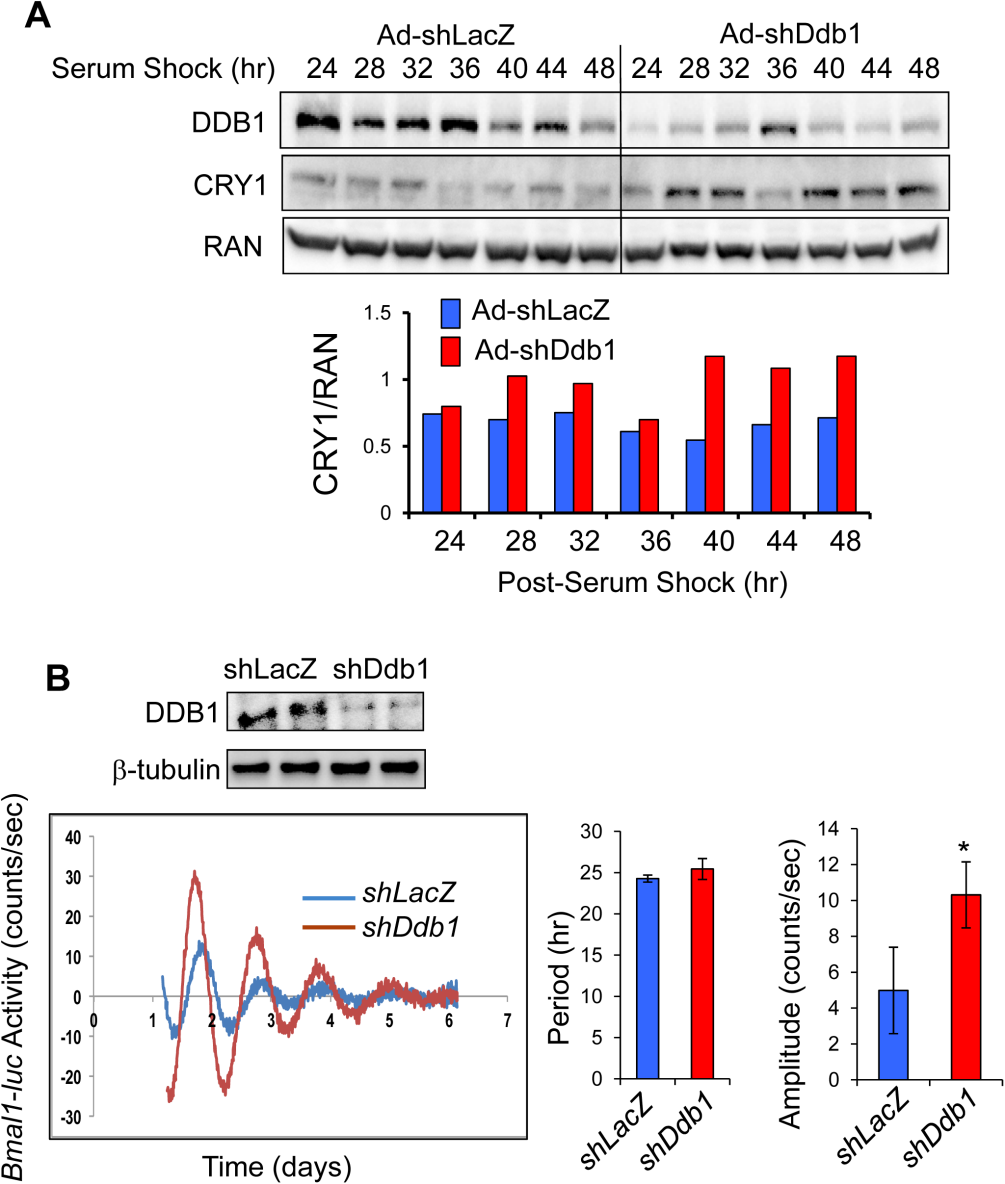
Knockdown of *Ddb1* enhances both circadian oscillations of CRY1 protein and *Bmal1* promoter-driven clock activity. (**A**) Depletion of *Ddb1* by adenoviral shRNA elevates CRY1 protein levels in Hepa1 cells. Mouse Hepa1 cells were transduced with Ad-shLacZ or Ad-shDdb1. 24 hr post-transduction, cells were synchronized by serum shock to reset circadian cycles. Protein samples were collected every 4 hr between 24 and 48 hr post-synchronization. The endogenous CRY1 and DDB1 levels were determined by immunoblotting. The protein level of RAN was measured as loading control. The levels of CRY1-FLAG were measured by immunoblotting, quantified, and normalized by the levels of loading control RAN (bottom panel). (**B**) Effect of acute *Ddb1* depletion on circadian activities of *Bmal1-luc* in U2OS cells. The *Bmal1-luc* U2OS stable cells were transfected with either shLacZ or shDdb1 prior to synchronization. Lumicycle monitored the oscillations of luciferase activities for five circadian cycles. Knockdown efficiency of *Ddb1* by shRNA was confirmed by anti-DDB1 immunoblotting in the top panel. Circadian period length and amplitude values were compared between shLacZ and shDdb1 groups (n = 4)**.** *p < 0.05 by the Student's *t*-test.

CRY1 protein levels have been shown to oscillate in a circadian manner [11]. To address whether CUL4A-DDB1 E3 ligase also regulates CRY1 protein oscillations in circadian cycles, we turned to an *in vitro* synchronization system that we established previously to study the molecular clock in Hepa1 hepatocytes [41]. We transduced Hepa1 cells with either Ad-shLacZ or Ad-shDdb1 and then synchronized the cells for a circadian study. We confirmed the knockdown efficiency by showing the reduced levels of DDB1 protein throughout the entire 24-hr period. In contrast with the depleted DDB1 level, the levels of CRY1 protein were elevated moderately at ZT (Zeitgeber time) 28 and 32 hr (< 1.5-fold) but drastically at ZT 40, 44, and 48 hr (> 1.5-fold) (**Fig 5A**). In summary, our data support that CUL4A-DDB1 function as a novel E3 ligase to control CRY1 protein oscillations during circadian cycles.

The temporal abundance of CRY1 protein determines the period length and amplitude of clock gene oscillations [11, 12, 42]. In the case of *Fbxl3* mutation, CRY1 protein is stabilized and maintains at a higher level throughout the circadian cycle, resulting in dampened expression of its target genes such as *Dbp* and *Per2 [12]*. We propose that depletion of *Ddb1* also leads to similar impact on the molecular clock activity via increased CRY1 protein abundance. To measure the effects of DDB1 on the circadian gene cycling, we employed the well-established U2OS stable cell line, which stably expresses the *Bmal1* promoter-driven luciferase and displays strong oscillations of luciferase activity after synchronization by dexamethasone and forskolin [31]. Surprisingly, we observed significantly increased amplitude of oscillations of the *Bmal1-luc* activity in *Ddb1*-depleted U2OS cells (**Fig 5B**).

**Fig 6:**
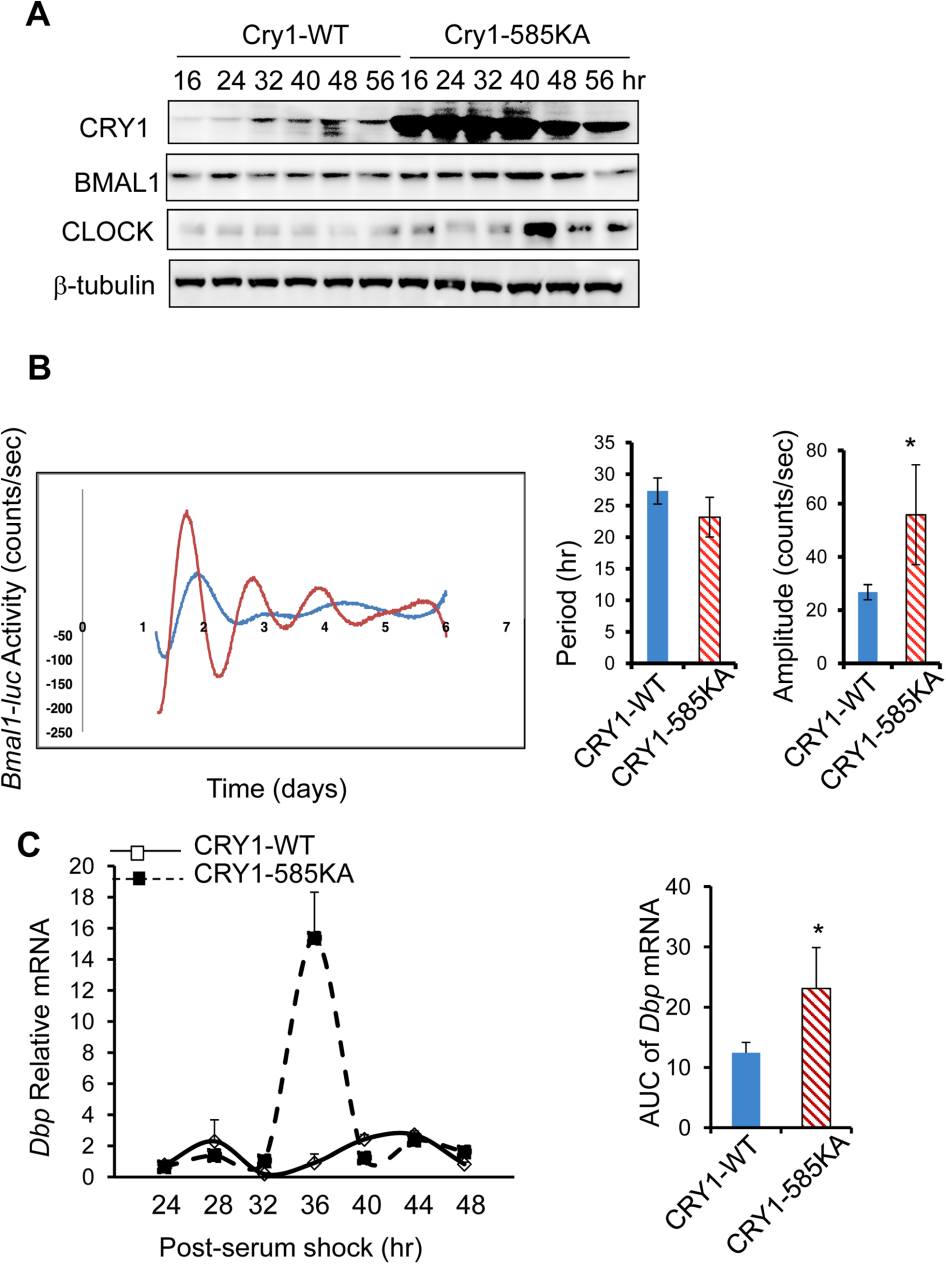
Impact of 585KA mutation on circadian oscillations of CRY1 protein, *Bmal1* promoter activity as well as clock output gene. (**A**) Circadian oscillations of CRY1-WT *vs*. CRY1-585KA in Hepa1 cells. 24 hr after transfection with Cry1 expression vectors (WT *vs*. 585KA), Hepa1 cells were synchronized by serum shock and harvested every 8 hr between 16 and 64 hr. The protein levels of CRY1 protein (WT *vs*. 585KA), BMAL1, and CLOCK were determined by immunoblotting with anti-FLAG or with protein-specific antibodies. (**B**) Impact of CRY1 WT or CRY585KA on oscillations of *Bmal1-luc* in U2OS cells. The *Bmal1-luc* U2OS stable cells were transfected with GFP, Cry1-WT, or Cry1-585KA and then subjected to Lumicycle analysis. The period length and amplitude values were calculated and compared. *p-value < 0.05 between CRY1-WT and Cry1-585KA group by the student’s *t*-test (n = 6). **(C)** Increased amplitude of the endogenous clock gene oscillations in cells overexpressing CRY1-585KA. U2OS cells were transfected with expression vectors encoding either Cry1-WT or Cry1-585KA. 24 hr post-transfection, cells were synchronized by serum shock and collected at the indicated time points. The endogenous *Dbp* mRNA levels were measured by RT-qPCR. The data were plotted as Mean ± S.D. (n = 4). The AUC (area under curve) analysis for *Dbp* mRNA oscillations was presented as well. * *p*-value < 0.05 by Student’s *t*-test.

To rule out the possibility that other DDB1-CUL4A E3 ligase substrates could contribute to the effects of *Ddb1*-knockdown on circadian oscillations, we tested the impact of the CRY1 585KA mutant on the molecular circadian clock activity since we showed that this mutant is resistant to regulation by DDB1-CUL4A E3 ligase (**Fig 3B and 3C**). Consistent with what we observed in 293T cells, CRY1-585KA maintains high levels of stability across all the time points in serum shock-synchronized Hepa1 cells in comparison with CRY1-WT protein (**Fig 6A**). Unexpectedly, we observed a concomitant increase in the endogenous levels of BMAL1 and CLOCK in Hepa1 cells expressing CRY1-585KA mutant. To assess the circadian effects of CRY1-585KA, we introduced either CRY1-WT or CRY1-585KA into U2OS cells stably expressing the *Bmal1-luc* reporter. In comparison with CRY1-WT, overexpression of CRY1 585KA mutant significantly enhances the amplitude of *Bmal1-luc* oscillations (**Fig 6B**), in agreement with the impact of *Ddb1* depletion on the *Bmal1-luc* activity (**Fig 5B**). To exclude the possibility that this CRY1 585KA mutant effect is *Bmal1* promoter-specific, we transfected U2OS cells with either CRY1 WT or 585KA and induced circadian synchronization by serum shock. In this setting, we observed more robust oscillations of the endogenous *Dbp* mRNA in cells expressing CRY1 585KA *vs*. CRY1 WT (**Fig 6C**). Taken together, our data show that either blocking DDB1-mediated CRY1 degradation or overexpressing 585KA mutation increases CRY1 protein stability and enhances the molecular clock oscillations.

## Discussion

In this report we discovered the DDB1-CUL4A-CDT2 complex as a novel E3 ligase targeting the mammalian CRY1 for ubiquitination and degradation to regulate its protein circadian oscillation and the circadian clock function. We provided both *in vitro* and *in vivo* evidence supporting that DDB1-CUL4A E3 ligase directly ubiquitinates CRY1 and regulates CRY1 stability. Our work identified CDT2 as the critical substrate receptor for mediating the DDB1-CUL4A E3 ligase activity on CRY1 protein ubiquitination and degradation. Furthermore, we mapped lysine 585 of CRY1 protein as the ubiquitination site for DDB1-CUL4A E3 ligase. Blocking DDB1-mediated CRY1 ubiquitination and degradation increases the amplitude of the molecular clock without altering the period length. In conclusion, our study revealed DDB1-CUL4A-CDT2 E3 ligase complex as a novel regulator of the mammalian CRY1 protein stability and the molecular clock function.

Based on most recent studies, a complex picture has emerged for the regulation of E3 substrates. Multiple E3 ligases can target the same or different residues of substrates for ubiquitination. Consequently, opposite effects are exerted on the protein function and stability in a signal-specific manner. For instance, the TrCP E3 ligase poly-ubiquitinates the c-Myc protein at the same residue as the FBXW7 E3 ligase via different linkage sites but protects c-Myc from degradation [43]. Similar to c-Myc, the tumor suppressor protein P53 was also shown to require a myriad of E3 ligases to control its turnover [44]. Our study demonstrated that CRY1 is another substrate that requires multiple E3 ligases to control its ubiquitination and degradation in response to various physiological signals and processes. AMPK has been shown to phosphorylate CRY1 at S71 of the N-terminus prior to FBXL3-dependent poly-ubiquitination in response to energy demand [14]. FBXL21 affects circadian rhythms by competing with FBXL3 to antagonize CRY1 degradation and block its nuclear localization [15]. FBXL21 targets a single residue at K107, while FBXL3 seems to target multiple lysines for ubiquitination [16]. Here, we have identified a novel E3 ligase targeting lysine 585 at the CRY1 C-terminal region, a completely different ubiquitination site than those used by FBXL3. Our results demonstrated that DDB1-CUL4A targets K585 for ubiquitination and a single lysine to alanine mutation at 585aa is sufficient to stabilize CRY1 protein against DDB1-CUL4A-mediated ubiquitination and degradation. Such regulation is independent of the AMPK-FBXL3 pathway since CRY1-S71A mutant still responds to DDB1-CUL4A-mediated ubiquitination and degradation as efficiently as CRY1-WT (**S2 Fig**). Thus, we revealed a novel ubiquitination pathway involving a completely different E3 ligase for CRY1 protein turnover during the circadian cycle. It will be of great interest to measure the *in vivo* effects of the slow-degrading CRY1-585KA mutant on the locomoter activity and sleep-wake cycles in a knock-in mouse model.

*Cdt2* encodes a 730-aa protein containing six highly conserved WD40-repeat domains and a consensus nuclear localization signal at the N-terminus. CDT2-containing CUL4A E3 ligase is known for its critical role in promoting cell cycle progression and preventing genome instability through ubiquitination and degradation of CDT1 [33], p21 [45], and Set8 [46] during the S phase of cell cycle and following DNA damage. Our work introduces a completely new function of CDT2 in degrading CRY1 protein. We have screened the tissue-specific expression profile of CDT2 in WT C57BL6 mice. Liver, adipose tissues, and spleen showed the highest CDT2 expression under both normal and diabetic conditions (unpublished data). Most recently, it has been reported that CRY1 stabilization mediates the effects of DNA damage on the circadian clock activity [47]. Thus, it is logical to speculate that CDT2 might couple the cellular metabolism with the DNA damage response by degrading CRY1 and other yet-to-be identified targets.

Our studies uncovered the unexpected impact on the molecular circadian clock when DDB1-mediated CRY1 degradation is blocked. Both acute depletion of *Ddb1* and over-expression of degradation-resistant *Cry1-585KA* mutant increase the amplitude but not the period length of the *Bmal1* promoter-driven clock activity, in contrast with increased CRY1 protein stability and dampened oscillations of clock genes in *Fbxl3*-deleted mice or *Fbxl3*-depleted cells [12, 42]. We suspect that diverging downstream molecular events may account for the different phenotypes in spite of general stabilization of CRY1 in all the cases. Possibly, FBXL3 mediates the basal turnover of CRY1 protein, whereas DDB1-CUL4A-CDT2 promotes signal-induced CRY1 degradation. Also, we speculate that CRY1 K585 residue may be modified via other post-translational modification mechanisms such as sumoylation, acetylation, or methylation in the case of DDB1 inhibition. These novel modifications may promote novel CRY1 protein interactions and stimulate unconventional circadian function. Of note, both BMAL1 and CLOCK protein levels are elevated in CRY-585KA-expressing 293T cells, suggesting that CRY1-585 mutant might enhance the clock activity by stabilizing both proteins through interactions with other clock components. An unbiased proteomic approach might be useful to answer all these questions in the future study.

## Supporting Information

S1 Fig CUL4A-DDB1-mediated CRY1 ubiquitination in Hepa1 and U2OS cells. (TIF)

S2 Fig CRY1 S71A mutant is still targeted by CUL4A-DDB1 E3 ligase for ubiquitination and degradation. (TIF)

S3 Fig Mapping the region of CRY1 targeted by CUL4A-DDB1 E3 ligase. (TIF)

S4 Fig Identification of CRY1-specific DCAF proteins through an shRNA screen. (TIF)
