# Supplementary figures and images for: CUL4-DDB1-CDT2 E3 Ligase Regulates the Molecular Clock Activity by Promoting Ubiquitination-Dependent Degradation of the Mammalian CRY1
Source: PLoS One. 2015 Oct 2;10(10):e0139725. doi: 10.1371/journal.pone.0139725 (PMC4592254; doi:10.1371/journal.pone.0139725)

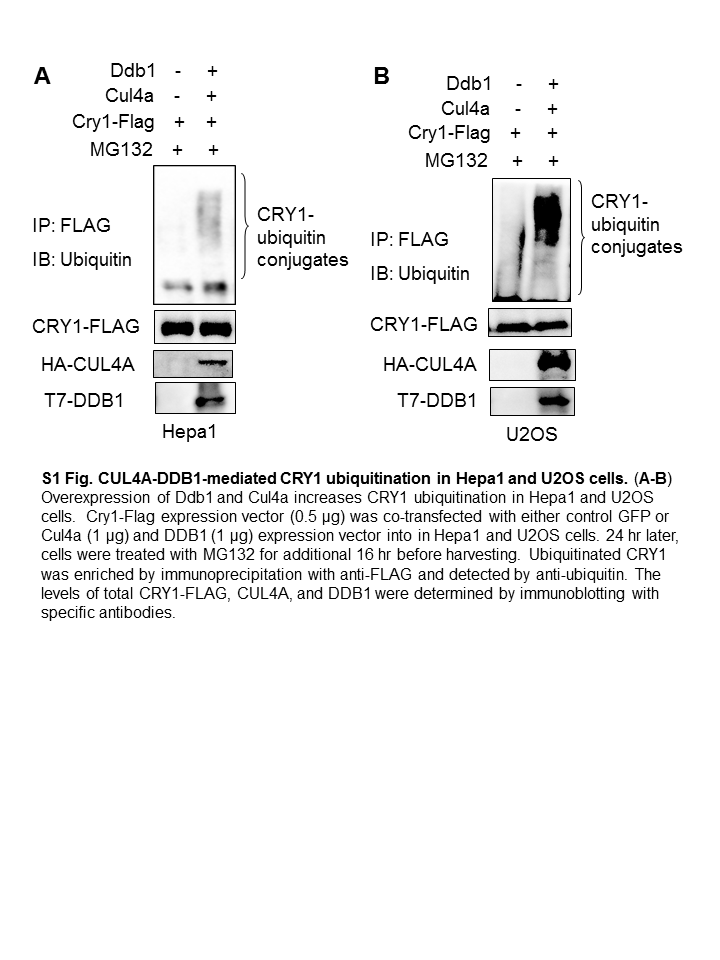

Supplement: S1 Fig — (TIF) [file pone.0139725.s001.tif]

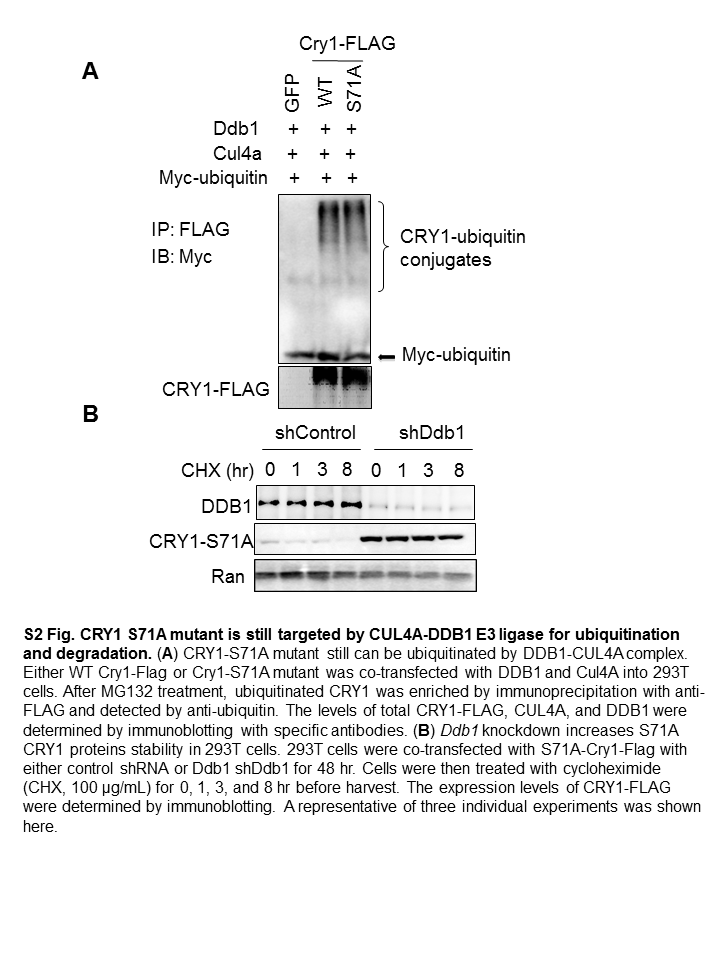

Supplement: S2 Fig — (TIF) [file pone.0139725.s002.tif]

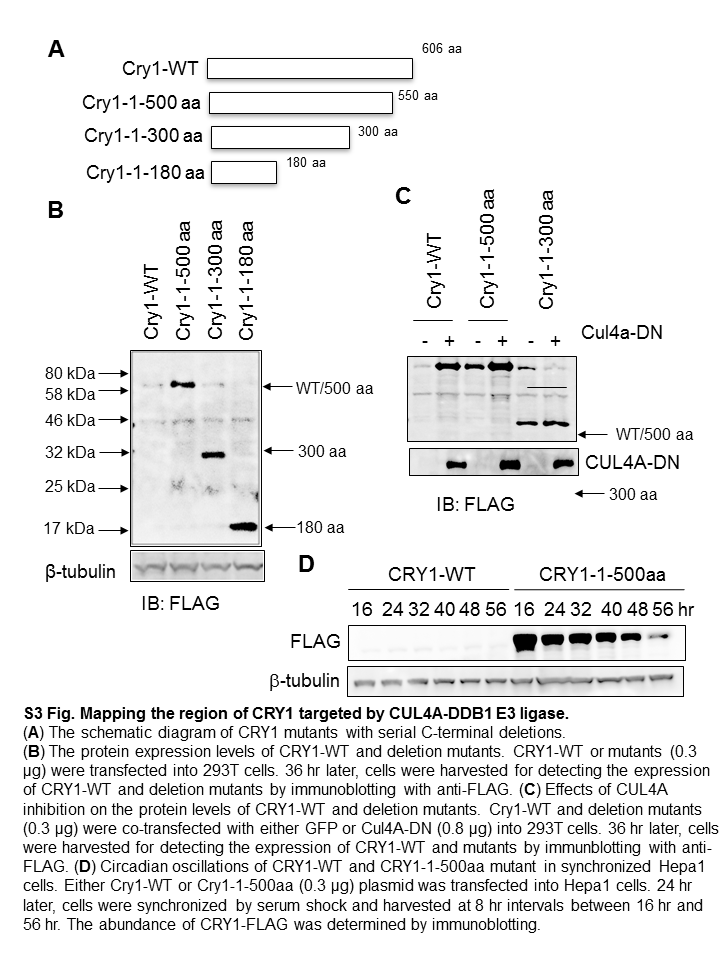

Supplement: S3 Fig — (TIF) [file pone.0139725.s003.tif]

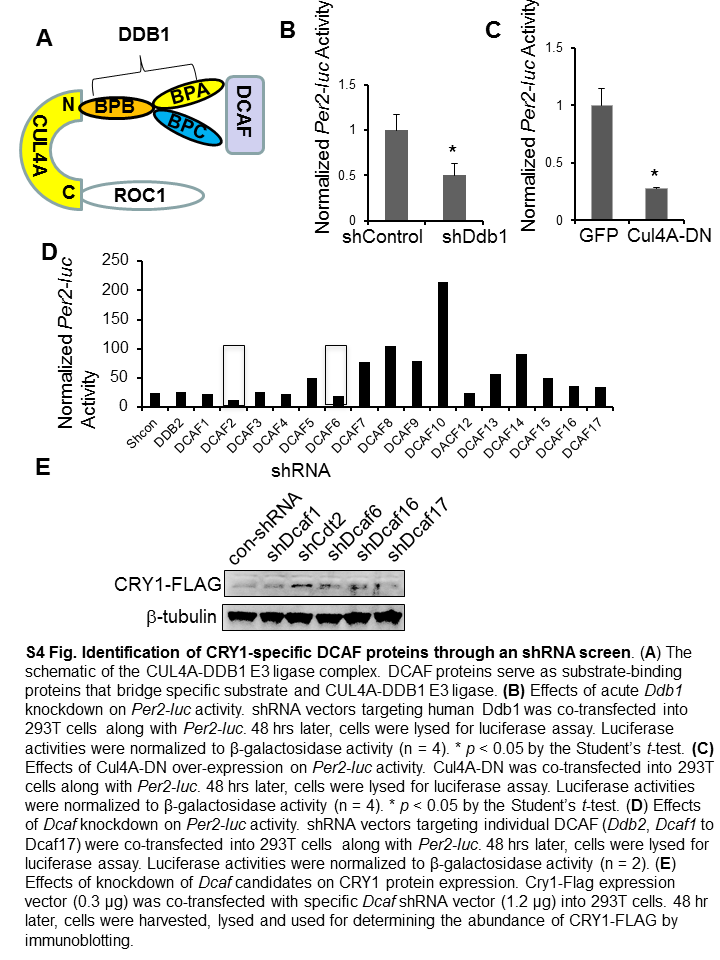

Supplement: S4 Fig — (TIF) [file pone.0139725.s004.tif]
